# Neonatal Epilepsy: Beyond Seizures in a Developing Brain—A Narrative Review

**DOI:** 10.3390/brainsci16060628

**Published:** 2026-06-11

**Authors:** Giovanni Boscarino, Eleonora Cresta, Lucia Leonardi, Maria Di Chiara, Alberto Spalice, Gianluca Terrin

**Affiliations:** 1 Department of Maternal and Child Health, Policlinico Umberto I, Sapienza University of Rome, 00161 Rome, Italy; eleonora.cresta@uniroma1.it (E.C.); lucia.leonardi@uniroma1.it (L.L.); maria.dichiara@uniroma1.it (M.D.C.); alberto.spalice@uniroma1.it (A.S.); gianluca.terrin@uniroma1.it (G.T.); 2 Pediatric Clinic, University Hospital, Department of Medicine and Surgery, University of Parma, 43126 Parma, Italy

**Keywords:** seizure, newborn, electroencephalogram, phenobarbital, immaturity, long-term outcome, neurodevelopment

## Abstract

**Highlights:**

**What are the main findings?**

- The immature neonatal brain generates a seizure phenotype characterized by electroclinical dissociation, with many seizures remaining electrographic-only and detectable only through continuous EEG monitoring.
- Etiology is the strongest determinant of outcome, while seizure burden, early EEG features, and treatment timing may independently influence long-term neurodevelopmental prognosis.

**What are the implications of the main findings?**

- Accurate management of neonatal seizures requires an individualized, etiology-driven approach integrating clinical assessment, continuous EEG, neuroimaging, and genetic testing.
- Emerging tools such as rapid genomic diagnostics, AI-assisted EEG analysis, and multimodal neuromonitoring may improve diagnostic precision, prognostic accuracy, and long-term neurodevelopmental outcomes.

**Abstract:**

Neonatal seizures represent the most common neurological emergency in the neonatal period and arise within a uniquely immature and highly dynamic brain. Their recognition is challenging due to frequent electroclinical dissociation, with many seizures remaining purely electrographic and therefore detectable only through continuous electroencephalogram (cEEG) monitoring. This narrative review provides an integrated and updated overview of neonatal seizures, bridging developmental neurobiology, diagnostic challenges, etiological classification, and therapeutic strategies. The immature brain is characterized by an imbalance between excitation and inhibition, transient network architectures, and activity-dependent developmental processes, all of which contribute to the distinct electroclinical features of neonatal seizures. cEEG remains essential for accurate diagnosis and quantification of seizure burden, which may influence outcome. Etiology represents the primary determinant of prognosis, with hypoxic–ischemic encephalopathy (HIE), stroke, and genetic disorders among the most frequent causes. Advances in genetic testing have improved diagnostic precision and enabled targeted therapies in selected cases, supporting a precision medicine approach. Several key findings emerge from the current evidence base: (i) the neonatal brain is a developmentally constrained system in which excitation–inhibition imbalance, transient circuits and immature long-range connectivity shape an electroclinically distinct seizure phenotype; (ii) cEEG is the gold standard for detection and quantification of seizure burden, since the majority of neonatal seizures are electrographic-only and bedside clinical recognition systematically underestimates true seizure burden; (iii) etiology—chiefly HIE, stroke, and genetic causes—remains the strongest determinant of outcome, while seizure burden acts as an independent and potentially modifiable prognostic modifier; (iv) phenobarbital retains an evidence-based advantage in acute electrographic seizure control, whereas levetiracetam offers a favorable safety profile in the absence of robust long-term human neurotoxicity data; (v) rapid genomic diagnostics, artificial intelligence-assisted EEG analysis and multimodal neuromonitoring are converging toward a precision-neonatology framework, but their translation into routine practice requires validation, standardization, and equitable access. Future neonatal seizure care should extend beyond seizure control to the preservation and optimization of neurodevelopmental outcomes.

## 1. Introduction

Neonatal seizures represent the most frequent neurological emergency in the neonatal period and a common manifestation of acute brain injury or, less commonly, underlying developmental disorders [1,2,3]. Their incidence is highest during the first days of life, reflecting the unique vulnerability of immature brain, which is characterized by evolving synaptic networks, a developmental imbalance between excitation and inhibition, and activity-dependent maturation processes [2,4]. In this context, seizures are not only a clinical sign but may also contribute to brain dysfunction, although their independent influence on long-term outcome remains incompletely defined [2,5,6].

Over the past decades, the clinical approach to neonatal seizures has undergone substantial transformation. The widespread adoption of continuous electroencephalogram (cEEG) monitoring has shown that a substantial proportion of seizures in neonates are electrographic-only, without overt clinical correlates [7,8]. This has challenged traditional diagnostic paradigms and raised critical questions regarding when and how seizures should be treated [7,9]. At the same time, advances in electroclinical classification and genomic diagnostics have expanded our ability to identify underlying etiologies, highlighting the heterogeneity of neonatal epilepsies and the limitations of a purely symptom-based approach [4,10,11,12].

Despite these advances, major gaps remain in the management of neonatal seizures. Therapeutic strategies still rely largely on historical antiseizure medications (ASMs) with limited efficacy, and high-quality neonatal-specific evidence remains scarce, although recent guidelines have strengthened the evidence framework [9,13,14]. Furthermore, the relationship between seizure burden, underlying etiology, and neurodevelopmental outcome is complex and not fully elucidated, complicating clinical decision-making [5,6].

In parallel, the field is increasingly moving toward a precision medicine paradigm, driven by the integration of electrophysiological, genetic, imaging, and molecular data [10,11,12,14]. However, in the neonatal context, this transition remains incomplete, and the translation of improved diagnostic capability into mechanism-based individualized treatment is still evolving [9,10,14].

A major conceptual challenge in this field is the distinction between neonatal seizures and neonatal epilepsy. Most neonatal seizures are acute symptomatic events occurring as a consequence of an identifiable brain insult, such as hypoxic–ischemic encephalopathy (HIE), perinatal arterial ischemic stroke, intracranial hemorrhage, central nervous system (CNS) infection, or metabolic derangement [2,3]. These seizures do not, by themselves, fulfill the International League Against Epilepsy (ILAE) operational definition of epilepsy [15]. In contrast, neonatal-onset epilepsies and developmental and epileptic encephalopathies (DEEs) arise from intrinsic epileptogenic substrates, including monogenic disorders, malformations of cortical development, and selected metabolic diseases [11,16]. Despite these differences, the diagnostic, electrographic, and therapeutic challenges encountered in the neonatal period frequently overlap [2,3].

Within this heterogeneous spectrum, DEEs—defined by the ILAE as conditions in which both the underlying etiology and the epileptic activity contribute to ongoing neurodevelopmental impairment [11]—occupy a central position. Among DEEs with onset within the first year of life, two age-of-onset windows are particularly relevant. The neonatal window includes KCNQ2-DEE, STXBP1-DEE, CDKL5-DEE and the broader category of early infantile DEEs as redefined in the 2022 ILAE syndromic classification [11]. The post-neonatal infantile window includes infantile epileptic spasms syndrome (West syndrome), typically presenting between 3 and 12 months of life and only exceptionally during the neonatal period, and Dravet syndrome (SCN1A-related), with onset most commonly between 4 and 8 months and rare neonatal presentations [11]. These syndromes are not the primary focus of this review, which is restricted to the neonatal period, but they form the immediate developmental continuum into which neonatal-onset seizures evolve and are referenced where pertinent.

In this narrative review, our specific aims are: (i) to synthesize the links between developmental neurobiology and the neonatal seizure phenotype; (ii) to critically appraise current diagnostic tools, with particular emphasis on the emerging role of integrated electroencephalogram (EEG) and genomic technologies; (iii) to examine current pharmacological treatments and their limitations, as well as emerging precision-medicine approaches targeting the molecular mechanisms of disease; and (iv) to explore future directions, including artificial intelligence (AI) applied to EEG, multimodal neuromonitoring, and biomarker development.

This narrative review was prepared in accordance with the Scale for the Assessment of Narrative Review Articles (SANRA) reporting recommendations [17]. A self-assessment of compliance with the six SANRA domains is provided in Supplementary Table S1 [17]. A literature search was conducted on PubMed/MEDLINE, Embase, Scopus, and the Cochrane Library. Search terms included “neonatal seizures”, “neonatal epilepsy”, “HIE”, “amplitude-integrated EEG”, “cEEG”, “genetic seizure”, “ASMs”, “phenobarbital”, “levetiracetam”, “precision medicine”, “serum biomarkers”, and “neurodevelopmental outcomes”. Reference lists of relevant articles and international guidelines were also reviewed to identify additional studies. Articles were selected based on their relevance and scientific contribution to the topics discussed, with particular attention to recent evidence and international recommendations, while also including seminal studies and landmark publications when appropriate.

Records were screened against predefined inclusion criteria, including original clinical studies, randomized controlled trials (RCTs), observational studies, systematic reviews, meta-analyses, international guidelines, position statements, and methodological review literature. Inclusion was restricted to articles addressing neonatal (≤44 weeks postmenstrual age) or directly translatable preclinical work, within the content domains of this review. Review articles were retained when considered useful for historical context or synthesis of evolving concepts. Publications not directly relevant to these topics were excluded. Two authors (G.B. and E.C.) independently screened the retrieved literature, and discrepancies were resolved through discussion with a third senior author (G.T.). Full texts were assessed by the same dual-review process. As a narrative review, no formal systematic review protocol was applied.

Several recurring themes emerge across the contemporary literature on neonatal epilepsy and provide a conceptual framework for understanding current advances and future directions in the field. These include: (i) developmental immaturity of the neonatal brain as a determinant of seizure expression and vulnerability; (ii) electroclinical dissociation as the central diagnostic challenge; (iii) etiology as the strongest determinant of outcome, with seizure burden acting as an independent modifier; and (iv) the integration of emerging technologies, including AI applied to EEG analysis, rapid genomic diagnostics, and multimodal monitoring, into neonatal neurological care.

## 2. The Neonatal Brain: A Developmental System

The neonatal brain is a highly dynamic and rapidly evolving system, characterized by transient developmental architectures that shape both physiological function and vulnerability to disease. Even at term, the brain should not be considered structurally or functionally complete, but rather a transitional state marked by ongoing synaptogenesis, dendritic arborization, myelination, and progressive large-scale network integration extending well beyond birth [18,19]. Early brain organization is dominated by activity-dependent processes and transient circuits, including the subplate zone, which play a crucial role in coordinating early cortical and thalamocortical connectivity [20,21]. These immature networks generate characteristic spontaneous activity patterns—such as delta brushes in preterm infants—that are essential markers of neural maturation and likely reflect early subplate-related network activity [22].

Following term birth, brain maturation continues at a rapid pace during the neonatal period and early infancy. Synaptogenesis accelerates significantly, with a rapid increase in synaptic density and dendritic complexity, particularly within cortical regions. In parallel, progressive myelination enhances signal transmission, while refinement of thalamocortical and cortico-cortical projections supports the emergence of more efficient and integrated networks. These maturational changes are paralleled by the transition from discontinuous to more cEEG activity and by the progressive organization of sleep–wake cycling (SWC), although functional specialization and hierarchical network architecture remain incomplete, indicating that even the term neonatal brain operates within a still-developing functional framework [18,23].

A key feature of early neurodevelopment is the progressive shift from predominantly local, short-range connectivity toward more efficient long-range integration. In preterm infants, this process is interrupted during a period of intense network construction and may result in fragmented functional organization and dysmaturation in an ex utero environment, with lasting effects on structural and functional connectivity [19,23,24]. At the cellular level, early neurophysiology is characterized by a relative predominance of excitation over inhibition, due in part to the depolarizing action of γ-aminobutyric acid (GABA) during early development, which promotes synchronization but also lowers seizure threshold [25].

**Figure 1:**
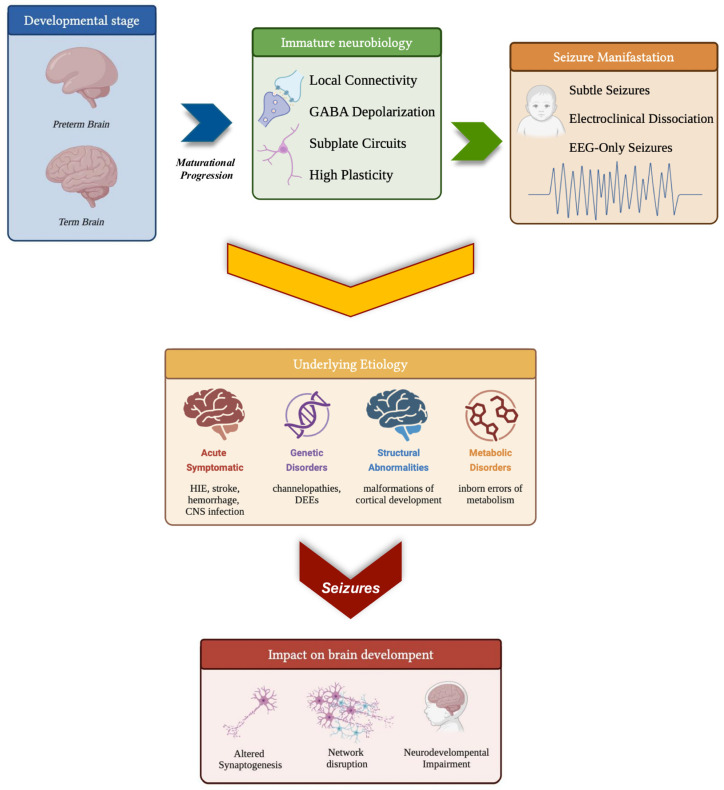
Developmental framework of neonatal brain and seizures. Figure Legend. In blue panel schematic representation not intended to reflect detailed anatomical cortical morphology. GABA (γ-Aminobutyric acid); EEG (electroencephalogram); HIE (Hypoxic–ischemic encephalopathy); CNS (Central nervous system); DEEs (Developmental and epileptic encephalopathies).

Within this developmental framework, neuronal activity is not merely a reflection of existing circuitry but a key driver of its maturation through tightly regulated activity-dependent mechanisms. Neonatal seizures emerge from developmentally constrained networks and, in turn, may interfere with, on the basis of experimental evidence in animals models [26], these physiological processes of brain maturation; whether this effect operates independently of the underlying brain injury in human neonates has not been established. The constraints are specific and quantifiable: (i) a still-active subplate zone that serves as the main early integrator of thalamocortical input before mature cortical circuits are in place [20,21]; (ii) immature thalamocortical and intracortical wiring with predominantly short-range, locally clustered connectivity [23,24]; (iii) sparse and slow long-range myelinated connections, contributing to a predominance of locally organized seizure activity and limiting the efficient propagation of activity across distributed cortical networks [23]; (iv) a developmental excitation–inhibition imbalance driven by the high NKCC1/KCC2 ratio and the resulting depolarizing action of GABA-A signaling [25]; and (v) immature SWC, so that the modulation of cortical excitability by behavioral state is not yet fully effective [22]. These developmental constraints likely contribute to the subtle, fragmented, and frequently subclinical semiology that characterizes neonatal seizures. This concept has critical implications for neonatal seizures, which should not be regarded solely as epiphenomena of underlying brain injury but also as potential modifiers of brain development (Figure 1).

Two mechanistically distinct situations should be kept separate when discussing the relationship between brain injury and seizures in the neonate. In acute symptomatic seizures—which account for the majority of cases—the primary insult damages the brain (HIE, perinatal arterial ischemic stroke, intraventricular or intracerebral hemorrhage, CNS infection), and seizures emerge as a downstream electrical manifestation of that injury, with the possibility that ongoing seizures further worsen secondary injury [3,7,11]. In intrinsically epileptogenic conditions, by contrast, the substrate itself generates seizures: this includes monogenic channelopathies, malformations of cortical development and inborn errors of metabolism (IEMs) [2,11]. The distinction matters clinically because the diagnostic priority, the expected therapeutic response and the prognostic counselling differ substantially between the two groups [2,3,7,11]. The patterns of regional vulnerability described below should therefore be read as a feature of the acute-symptomatic group.

Experimental evidence indicates that excessive or dysregulated neuronal activity during sensitive developmental windows can disrupt synaptogenesis, alter dendritic growth, impair circuit refinement, and promote maladaptive network organization [26]. These physiological characteristics are closely intertwined with patterns of brain injury in the neonatal period. In preterm infants, vulnerability is greatest in the periventricular white matter, where immature oligodendrocyte lineage cells are particularly susceptible to hypoxic–ischemic and inflammatory insults, contributing to impaired connectivity development and dysmaturation [19,27]. In contrast, in term neonates, injury more commonly involves the deep gray nuclei and cortical regions, reflecting maturational differences in cerebral perfusion and metabolic demand [28]. Importantly, such injuries do not simply produce static lesions but may alter ongoing developmental programs and network trajectories [19,27,28].

These developmental and pathological features are directly reflected in the semiology of neonatal seizures. Seizures are often subtle, fragmented, and poorly lateralized, and a defining characteristic is the frequent dissociation between electrographic and clinical manifestations, with many seizures remaining exclusively electrographic [7,29]. This electroclinical mismatch reflects the intrinsic immaturity of neural networks and their limited capacity to generate coordinated motor outputs through still-developing corticospinal and corticocortical systems [20,21,23].

Moreover, limited myelination and inefficient long-range connectivity further restrict propagation of epileptic activity across distributed networks, favoring the occurrence of subclinical or purely electrographic seizures [7,23]. This electroclinical dissociation may be further amplified by (ASMs), which can suppress clinical manifestations without fully abolishing the underlying electrical seizure activity, making cEEG monitoring essential for accurate diagnosis and management in this population [29,30,31]. As brain maturation progresses, the epileptic phenotype evolves accordingly, transitioning toward more organized and electro-clinically correlated seizure types in later infancy and childhood [11].

Advances in multimodal neurophysiology and neuroimaging have provided important insights into these processes. cEEG remains the gold standard for detecting neonatal seizures and for characterizing age-specific background activity, including a substantial burden of subclinical events [7,30,31]. Complementary techniques such as diffusion magnetic resonance imaging (MRI), functional MRI, and magnetic resonance spectroscopy allow in vivo assessment of structural connectivity, functional network organization, and cerebral metabolism in the developing brain [9,28,32]. This integrated perspective underscores the need to interpret neonatal seizures within the broader context of brain development, with important implications for early diagnosis, prognostication, and targeted therapeutic strategies.

## 3. From Genotype to Phenotype

The correlation between genotype, EEG patterns, and therapeutic response represents one of the most tangible applications of precision medicine in neonatal epilepsies, although it remains incomplete [11,16,33].

Among genetic epilepsies with neonatal onset, ion channelopathies—particularly those involving KCNQ2, KCNQ3, and SCN2A—represent one of the clearest examples of genotype–phenotype correlation. These disorders link specific molecular dysfunctions of ion channels to recognizable electroclinical patterns and, in selected cases, to targeted therapeutic strategies. This paradigm has become a cornerstone of precision medicine in neonatal epilepsies, where early genetic diagnosis can directly inform treatment choices and improve clinical management [11,34,35].

A distinctive electroclinical signature has been described in these disorders, characterized by asymmetric tonic posturing followed by clonic movements and autonomic changes, often referred to as sequential seizures [7,16]. On EEG, this pattern is associated with an initial electrical decrement followed by rhythmic spike activity and postictal suppression, while amplitude-integrated EEG (aEEG) typically shows a rapid increase in amplitude followed by depression [16,35]. Recognition of these electroclinical features is clinically relevant, as it may suggest an underlying genetic channelopathy and support early diagnostic and therapeutic decisions [11,16,33].

In this context, epilepsies related to KCNQ2 and SCN2A variants frequently respond to sodium channel blockers, such as carbamazepine, phenytoin, and oxcarbazepine, particularly in early-onset gain-of-function conditions [16,36,37]. Importantly, the relationship between genotype and treatment response depends not only on the gene involved but also on the specific functional effect of each variant. In SCN2A-related epilepsies, gain-of-function variants are typically associated with neonatal or early infantile onset and show a favorable response to sodium channel blockers, whereas loss-of-function variants are more often linked to later-onset phenotypes and may show limited or absent response, or even worsening with these treatments [36,37,38].

A symmetrical pattern emerges for KCNQ2, the other channelopathy central to the neonatal-onset spectrum. Loss-of-function and dominant-negative variants—the most frequent disease mechanism—typically produce severe early-onset KCNQ2 encephalopathy, with burst-suppression EEG, neurological depression in the first days of life, and limited or absent response to standard ASMs [35]. By contrast, the less frequent gain-of-function variants can underlie phenotypes that respond favorably to sodium channel blockers, in particular carbamazepine, as illustrated by the Dilena case report [16]. In KCNQ2 as in SCN2A, therefore, the functional direction of the variant—not merely the gene involved—predicts therapeutic response. This bidirectional principle is the strongest available argument for adding early functional characterization of variants to genetic identification in the neonatal precision-medicine workflow. Across KCNQ2-, KCNQ3-, and SCN2A-related epilepsies, the functional consequence of the variant is at least as important as the causal gene itself in determining treatment response. Gain-of-function variants in KCNQ2 and SCN2A generally show favorable responses to sodium-channel blockers, whereas loss-of-function or non-missense variants are typically associated with developmental and epileptic encephalopathy phenotypes, limited or absent treatment response, and a less predictable relationship between genotype and therapy [39,40]. In addition, most available evidence derives from KCNQ2, and therapeutic recommendations are often extrapolated to KCNQ3 despite the much smaller published literature [41]. Consequently, genotype-guided treatment should not be reduced to gene identity alone but should, whenever possible, incorporate functional characterization of the specific variant [39,40].

Beyond ion channelopathies, two further genes are increasingly recognized as central to the neonatal-onset DEEs spectrum and merit explicit discussion. STXBP1-related encephalopathy typically presents in the neonatal or early infantile period with tonic seizures, often associated with a burst-suppression background and severe developmental delay [42]. Seizures may show a partial response to levetiracetam and to high-dose vitamin B6 in selected cases, but the developmental component dominates the long-term phenotype [42]. CDKL5 deficiency disorder, X-linked dominant with female predominance, manifests as early-onset epileptic encephalopathy with multifocal seizures and frequent evolution toward infantile epileptic spasms; response to conventional ASMs is typically poor and a positive allosteric modulator of GABA-A receptors (ganaxolone) has recently been approved as the first disease-targeted treatment [43]. In both conditions, as for KCNQ2 and SCN2A, early molecular diagnosis directly informs treatment choices and prognostic counselling [11].

Although single-gene epilepsies and IEMs may present with similar neonatal seizure phenotypes, they represent biologically distinct disease categories. Monogenic DEEs, including KCNQ2-, SCN2A-, STXBP1-, and CDKL5-related disorders, arise primarily from abnormalities of neuronal excitability, synaptic transmission, or neurodevelopmental pathways [11]. In contrast, metabolic epilepsies, such as Aldehyde dehydrogenase 7 family member A1 (ALDH7A1)-related pyridoxine-dependent epilepsy, PNPO deficiency, non-ketotic hyperglycinemia, and sulphite oxidase or molybdenum cofactor deficiencies, result from disruption of specific biochemical pathways [2]. This distinction has important clinical implications, as several metabolic epilepsies are potentially treatable and require urgent recognition to prevent irreversible neurological injury [2]. Early differentiation between these entities therefore informs both diagnostic prioritization and therapeutic decision-making in the neonatal period. EEG alone therefore cannot distinguish these two groups in the neonatal period; rapid metabolic and genetic workup is required.

Malformations of cortical development add a third mechanistic axis to the genotype–phenotype landscape of neonatal-onset epilepsy. In tuberous sclerosis complex (TSC1/TSC2), the structural substrate is given by cortical tubers; seizures most commonly start in the first months of life—and rarely already in the neonatal period—as focal events that frequently evolve into infantile epileptic spasms syndrome. Vigabatrin is established as first-line therapy and mTOR inhibitors (everolimus, sirolimus) are increasingly used as targeted treatments [44]. In LIS1-related lissencephaly, severe early-onset epilepsy is the rule and the response to conventional ASMs is typically poor, with multifocal interictal discharges on EEG and frequent evolution into infantile spasms [45]. Compared with monogenic channelopathies and with IEMs, the seizure semiology in malformations of cortical development is more often multifocal from the outset, the EEG more often shows multifocal rather than burst-suppression patterns, and the therapeutic decision tree is dominated by the structural substrate [2,11].

The principal genetic epilepsies presenting in the neonatal period and their major diagnostic and therapeutic implications are summarized in Table 1.

Despite these advances, the application of precision medicine in the neonatal period remains inherently limited. In many cases, treatment decisions must be made before genetic results are available, and functional characterization of variants is rarely accessible in real time. As a result, clinicians often rely on electroclinical features—such as specific EEG patterns—to infer the underlying etiology and guide therapy, highlighting both the progress made and the current gap between genetic knowledge and its translation into bedside care [11,47,48].

### Same Gene, Different Phenotypes

A key limitation of genotype-based approaches in neonatal epilepsy lies in the dynamic nature of brain development, which critically shapes the functional impact of genetic variants across different stages of life [48,49]. During the neonatal period, the brain is characterized by rapidly evolving neuronal networks, immature synaptic organization, and a predominance of excitatory mechanisms, all of which influence seizure generation and propagation [33,50]. In particular, the expression and function of ion channels undergo significant developmental changes, with shifts in channel subtypes, distribution, and biophysical properties over time. For example, sodium channel isoforms such as Nav1.2 and Nav1.6 show age-dependent expression patterns, which may alter the electrophysiological consequences of SCN2A variants across development [36,49]. As a result, the same genetic variant may produce different functional effects depending on the developmental context in which it is expressed.

This developmental modulation contributes to the marked phenotypic variability observed in genetic epilepsies, where the same gene—and even the same variant—can be associated with a spectrum ranging from self-limited neonatal seizures to severe DEEs or later-onset epilepsy syndromes [34,35,36].

In addition, the balance between excitatory and inhibitory neurotransmission evolves substantially during early life, with depolarizing γ-aminobutyric acid-mediated (GABAergic) signaling in the neonatal brain further amplifying network excitability and potentially modifying the clinical expression of genetic epilepsies [33,50].

Taken together, the factors emerging from this synthesis—the developmental modulation of voltage-gated ion-channel expression, with shifts in Nav1.2 and Nav1.6 distribution over time [49]; the depolarizing action of GABA-A signaling driven by the NKCC1 > KCC2 ratio in the immature brain [25]; and the still-sparse and immature long-range connectivity that limits propagation of epileptic activity [23]—indicate that genotype alone is insufficient to predict the clinical phenotype in neonatal epilepsy. The developmental stage in which a variant is expressed shapes its electroclinical and therapeutic translation, and a developmental perspective is therefore essential to any precision-medicine approach in this population [47,48].

## 4. Diagnostic Challenges in Neonatal Seizures

The diagnosis of neonatal seizures relies fundamentally on EEG yet remains intrinsically complex and often imprecise [9,51]. Neonatal EEG is characterized by marked physiological variability, discontinuous background activity, immature sleep–wake cycling (SWC), and age-dependent graph elements that complicate interpretation and may obscure the distinction between normal developmental patterns and pathological abnormalities [9].

A central limitation of neonatal seizure diagnosis is the dissociation between electrographic activity and clinical manifestations [9,52]. Neonatal seizures are frequently subclinical, and even when clinical signs are present, they are often subtle, brief, and difficult to recognize [9,52]. In a study using continuous video-EEG monitoring, only approximately one-third of electrographic seizures had any observable clinical correlate, and less than 10% were correctly identified by clinical staff [53]. This discrepancy demonstrates that clinical observation alone systematically underestimates the true seizure burden [52,53]. Furthermore, electroclinical uncoupling—particularly following administration of ASMs—can further obscure recognition, as electrographic seizures may persist despite apparent clinical resolution [31,52].

Conversely, overdiagnosis represents an equally important challenge. Neonates frequently exhibit paroxysmal motor or autonomic events that mimic seizures but lack an epileptic correlate on EEG [9,52]. These include jitteriness, benign sleep myoclonus, and brainstem release phenomena, which are commonly misinterpreted as epileptic events [52]. Indeed, a substantial proportion of clinically suspected seizures have no EEG correlate, leading to unnecessary treatment and potential exposure to ASMs [9,53]. In addition, interobserver agreement in clinical seizure recognition is poor, particularly for non-clonic events, further limiting the reliability of bedside assessment [52].

cEEG monitoring has therefore emerged as the gold standard for neonatal seizure detection [3]. It enables accurate identification of electrographic seizures, quantification of seizure burden, and evaluation of treatment response [52]. Importantly, seizure burden measured by cEEG has been consistently associated with adverse neurological outcomes, including increased mortality and neurodevelopmental impairment [9,29]. Despite its recognized value, the implementation of cEEG remains limited by resource availability, the need for specialized expertise, and challenges in continuous data interpretation [9].

aEEG has emerged as a pragmatic bedside tool in many neonatal intensive care units (NICUs) settings [54]. While useful for background trend analysis and initial screening, aEEG has reduced sensitivity compared to conventional multichannel EEG, particularly for brief, focal, or low-amplitude seizures [31,52]. As such, it should be considered complementary rather than equivalent to full EEG, especially when diagnostic precision is required [31,52].

It is useful to define the two techniques operationally. Conventional video-EEG (and its prolonged form cEEG) uses a full neonatal montage (≥11 scalp electrodes following the 10–20 system adapted for neonates [55]) with synchronized video recording and provides spatial resolution sufficient for detection of focal and brief electrographic events, characterization of background activity, and accurate quantification of seizure burden [31,55]. aEEG is derived from one or two channels through filtering, rectification and time compression, producing a semi-logarithmic display of upper and lower amplitude margins [54]. aEEG was designed for continuous bedside monitoring by neonatologists and not as a diagnostic substitute for full EEG [54]. In the typical NICUs workflow, aEEG is the preferred tool when (i) trained neurophysiology coverage is unavailable around the clock, (ii) the clinical question is screening of high-risk neonates (HIE undergoing therapeutic hypothermia, critically ill preterm infants, suspected sepsis with encephalopathy) rather than confirmation of focal events, and (iii) continuous trend assessment of background activity (continuity, SWC) is more informative than spatial detail [2,9,54,56]. cEEG should be preferred whenever diagnostic precision is required—for instance to confirm and localize focal seizures, to quantify seizure burden for treatment escalation, or in any neonate with persistent neurological signs and inconclusive aEEG. In our practice, aEEG and cEEG are complementary rather than competing modalities; the most realistic neonatal neuromonitoring model in most centers combines bedside aEEG with on-demand cEEG and remote expert review.

Although seizure semiology alone is often insufficient to establish the underlying cause, certain electroclinical patterns and associated investigations may help guide the diagnostic work-up. Table 2 summarizes the key diagnostic clues, EEG characteristics, and therapeutic implications across the major etiological categories of neonatal seizures.

Beyond neurophysiological monitoring, emerging evidence suggests that biochemical biomarkers may contribute to improving diagnostic accuracy [57]. Circulating markers of brain injury and oxidative stress have been proposed as adjunctive tools for early detection and risk stratification, potentially complementing EEG findings in the future [57]. However, their clinical utility remains investigational, and integration into routine practice is limited [57].

Taken together, these considerations highlight a critical diagnostic paradox: neonatal seizures are both underdiagnosed and overdiagnosed [9,51]. Subclinical seizures and electroclinical dissociation lead to systematic underestimation of seizure burden, while misinterpretation of non-epileptic events contributes to overtreatment [9,52,53]. This imbalance reflects not only technical and logistical limitations but also the unique neurobiology of the neonatal brain, in which seizure expression differs fundamentally from later developmental stages [9,51].

Despite advances in neurophysiological monitoring and growing interest in multimodal approaches, neonatal seizure diagnosis remains an imperfect process [9,51]. Improving diagnostic accuracy will require broader implementation of cEEG, integration of complementary tools, standardized interpretation frameworks, and a deeper understanding of neonatal brain physiology [9,51,57].

In addition, the distinction between epilepsy and encephalopathy in the neonatal period remains a critical and unresolved issue [9,31]. In this context, seizures are often identified in a brain that is already structurally and functionally compromised, making it difficult to determine whether they represent the primary pathological process or rather a secondary manifestation of an underlying encephalopathy [7,58]. In neonates, seizures rarely occur as an isolated disorder but are most commonly part of a broader clinical picture characterized by hypoxic–ischemic injury, intracranial hemorrhage, metabolic disturbances, or genetic disorders, all of which may independently impair brain function [29,58]. This overlap makes it difficult to distinguish between epilepsy—defined as a disorder of recurrent unprovoked seizures—and encephalopathy, in which global brain dysfunction may itself predispose to epileptic activity [15,59]. As a result, seizures may represent both a symptom of underlying brain injury and a potential contributor to neurological damage [26,29].

The concept of DEEs, introduced by the ILAE, provides a conceptual framework to address this complexity [59]. DEEs recognizes that neurological impairment may arise from two partially independent but interacting mechanisms: the direct effect of the underlying etiology on brain development (developmental component) and the additional deleterious impact of epileptic activity (epileptic component) [11,59]. Importantly, the relative contribution of these two factors is not fixed but exists along a continuum, which is particularly difficult to delineate in the neonatal period [11,48].

Clinical studies have shown that a higher seizure burden is associated with worse neurodevelopmental outcomes, even after adjusting for etiology, supporting a potential role of epileptic activity in exacerbating neuronal damage [9,29]. Experimental data indicate that excessive synchronous neuronal activity during critical periods of brain maturation may interfere with synaptogenesis, disrupt emerging network architecture, and alter activity-dependent plasticity [26]. Seizures may therefore act as a biomarker of disease severity rather than a direct driver of injury [9]. This issue is particularly evident in acute symptomatic seizures, such as those associated with HIE, where both the underlying injury and the epileptic activity evolve dynamically and influence each other [29,60].

Conversely, in several neonatal epilepsies—especially those of genetic origin—the encephalopathy may precede seizure onset and largely determine the clinical phenotype. In these conditions, early neurological impairment can be observed even before the emergence of seizures, suggesting that epileptic activity represents only one manifestation of a broader developmental disturbance [11,61]. This is particularly relevant in monogenic disorders, including channelopathies, where the same genetic variant can produce different phenotypes depending on the developmental stage, highlighting the strong interaction between genotype and brain maturation [34].

Rather, seizures should be conceptualized as both a marker and a potential mediator of disease severity, whose impact varies depending on the underlying etiology, developmental stage, and seizure burden [11,29].

### 4.1. Focus on EEG

The acquisition of neonatal EEG requires strict technical standards to ensure reliable assessment of brain function. A neonatal EEG system consists of scalp electrodes connected to a high-quality amplifier and a digital acquisition system, with appropriate hardware filtering and a sampling rate of at least 256–512 Hz to avoid signal distortion and aliasing [55]. Continuous synchronized video recording is essential, as it allows correlation between electrographic activity and clinical events, improving diagnostic accuracy [31,55]. Electrode placement should follow the international 10–20 system adapted for neonates, with a minimum of 11 electrodes to ensure adequate spatial resolution and interhemispheric comparison [55].

Neonatal EEG should be performed as a polygraphic recording [55]. In addition to cerebral activity, at least electrocardiographic and respiratory signals must be simultaneously acquired, while electrooculography, electromyography, and oxygen saturation monitoring can further enhance interpretation [55]. This multimodal approach is crucial for distinguishing true epileptic activity from physiological or environmental artefacts, which are particularly frequent in neonatal recordings [55,62]. The recording must be conducted under controlled environmental conditions, minimizing external stimuli such as noise and light, and adhering to developmental care principles to avoid disturbing the infant’s physiological state. Strict aseptic precautions and careful handling are mandatory due to the fragility of neonatal skin and the associated risk of infection [55]. Given the technical complexity of neonatal EEG, both acquisition and monitoring require trained personnel with specific expertise [55]. Adequate recording duration is critical for meaningful interpretation. A minimum of one hour is generally required to capture representative background activity, although recording a complete sleep–wake cycle is preferable. In high-risk or critically ill neonates, prolonged or cEEG monitoring—up to 24 h or longer—may be necessary to detect intermittent or subclinical seizures and to accurately quantify seizure burden [31].

Neonatal EEG interpretation should follow a systematic and stepwise approach, as it is highly age-dependent and requires integration of gestational age, postmenstrual age, clinical context, medications, and recording conditions [31,55]. The normal neonatal EEG reflects the progressive maturation of cortical networks, with characteristic changes in continuity, amplitude, synchrony, and organization over time [62,63]. Gestational age represents one of the most important determinants of EEG features (Table 3), with a transition from highly discontinuous patterns in extremely preterm infants to predominantly continuous and organized activity at term [62,63]. Importantly, neonatal EEG features vary continuously with postmenstrual age and are modulated by sleep state, medications (sedatives, ASMs, neuromuscular blockers), illness severity (sepsis, hypothermia, hypoglycemia) and recording quality.

This maturation is characterized by increasing continuity, interhemispheric synchrony, and spatial organization of background activity, as well as the emergence of age-specific graphoelements such as delta brushes and encoches frontales (Table 3) [62,63].

The discrete gestational age bands shown in Table 3 are a teaching simplification and should not be used as a binary normal/abnormal classifier in an individual neonate; clinical interpretation requires integration with polygraphic signals and the clinical context [55,62,63]. Interpretation must always consider vigilance states, as SWC is a key marker of functional brain maturation [62,63]. The first step in EEG interpretation is a global assessment of background activity, including continuity, amplitude, symmetry, synchrony, and spatial organization [55]. These features provide essential diagnostic and prognostic information, particularly in preterm or critically ill neonates [31,55]. The second step is the identification of vigilance states by integrating EEG findings with polygraphic signals and synchronized video recording [55]. The third step involves detailed analysis of physiological and pathological patterns, including morphology, frequency, amplitude, distribution, and temporal evolution [55].

Artefact recognition represents a critical component of neonatal EEG interpretation, as artefacts are extremely frequent and may closely mimic electrographic seizures [55,62,64]. Neonatal EEG is particularly vulnerable to artefacts due to the low amplitude and discontinuous background activity, as well as the complex intensive care environment [62,64]. Artefacts can be broadly classified into technical and biological sources. Technical artefacts include electrical interference, poor electrode contact, and environmental devices, whereas biological artefacts include muscle activity, body movements, eye movements, sucking, cardiac pulsations, and respiratory activity [64]. Repetitive or rhythmic artefacts may be difficult to distinguish from true seizures, particularly in preterm infants where physiological patterns are inherently variable [62,64]. A key distinguishing feature of electrographic seizures is their evolution over time in frequency, amplitude, or spatial distribution, whereas artefacts typically lack consistent spatiotemporal evolution and often correlate with identifiable physiological or environmental events [55,63].

Accurate interpretation therefore requires a multimodal approach integrating raw EEG, polygraphic signals, and synchronized video recording [31,55]. Misinterpretation of artefacts may lead to both overdiagnosis and underdiagnosis of neonatal seizures, with important clinical consequences [7,55]. According to the ILAE neonatal seizure framework, an electrographic seizure is defined as a sudden, repetitive, evolving, and stereotyped EEG pattern with a clear beginning and end, showing evolution in frequency, morphology, amplitude, or spatial distribution [7]. Conventional video-EEG remains the gold standard for confirming neonatal seizures, quantifying seizure burden, defining seizure onset and propagation, and correlating electrographic activity with clinical manifestations [31,55].

### 4.2. Focus on Amplitude-Integrated EEG

aEEG is a simplified method for continuous bedside monitoring of cerebral function in neonates and is widely used in NICUs for both background assessment and seizure screening. The technique is derived from conventional EEG through signal filtering, rectification, and time compression, resulting in a semi-logarithmic display of the upper and lower amplitude margins over time. Interpretation of aEEG is primarily based on the assessment of background activity, which reflects the functional integrity and maturation of the neonatal brain [65,66].

From a clinical perspective, aEEG is mainly used in neonates at high risk of brain injury or seizures, including those with HIE, suspected or confirmed neonatal seizures, prematurity with risk of cerebral complications, CNS infections, and critically ill infants requiring neuromonitoring [31,54,56,67,68]. In these settings, aEEG allows early identification of abnormal background patterns, monitoring of disease evolution, and continuous screening for electrographic seizures [66,67,68].

The aEEG background is typically classified into distinct patterns, including continuous normal voltage (CNV), discontinuous normal voltage (DNV), burst suppression (BS), continuous low voltage (CLV), and flat trace (FT). CNV is characterized by a continuous trace with a lower margin generally above 5 µV and is considered normal in term neonates, whereas DNV may be physiological in preterm infants. Severely abnormal patterns such as BS, CLV, and FT are associated with significant cerebral dysfunction and adverse neurological outcomes. In addition, the presence of SWC, visible as cyclic variations in bandwidth, provides further information on brain maturation and is often absent or disrupted in encephalopathic neonates [65,66]. Interpretation of aEEG is particularly challenging in preterm infants, in whom background activity is physiologically discontinuous, of lower amplitude, and characterized by immature or absent SWC [65]. These developmental features may overlap with pathological patterns and reduce the reliability of background classification [31,65,66,68].

Seizure activity on aEEG is typically identified by a sudden increase in both the upper and lower margins of the trace, resulting in a characteristic widening of the bandwidth. Repetitive seizures may produce a stereotyped “saw-tooth” pattern. However, the sensitivity of aEEG for seizure detection is limited and depends on seizure duration, amplitude, and spatial extent. Prolonged and generalized seizures are more likely to be detected, whereas brief, low-amplitude, or focal seizures may be missed [67,68,69]. Recent systematic reviews confirm that aEEG is less accurate than conventional video-EEG/cEEG for neonatal seizure detection, and current American Clinical Neurophysiology Society guidance supports cEEG as the gold standard for diagnosis and quantification of neonatal seizures [31,69,70]. Table 4 shows a comparison between aEEG and conventional EEG in neonatal seizure monitoring. Comparative studies indicate that aEEG detects on average approximately 40–60% of electrographic seizures identified on conventional multichannel EEG, but this figure should be read as a population-level mean that hides important effect-modifier heterogeneity. Reported sensitivity varies with the number of channels (single-channel C3–C4 vs. dual-channel C3–P3/C4–P4), seizure duration (events shorter than ~30 s are frequently missed), spatial extent (focal and low-amplitude events particularly susceptible to under-detection), background activity, interpreter experience, and whether raw EEG segments are reviewed alongside the compressed trace [67,68,69]. The recent Cochrane systematic review by Rakshasbhuvankar et al. [51] formally documents this between-study heterogeneity, with individual-study sensitivity ranging from approximately 31% to 90% depending on technique and reader. Consequently, although aEEG is a valuable tool for continuous monitoring and detection of a high seizure burden, it underestimates total seizure activity compared with conventional EEG (Table 4).

## 5. Therapy: Fragile Evidence in Neonatal Epilepsy

The treatment of neonatal seizures remains one of the most controversial areas in pediatric neurology, characterized by a substantial gap between clinical practice and high-quality evidence. Despite decades of use, therapeutic strategies are still largely based on historical precedent, small observational studies, and extrapolation from older populations, rather than robust RCTs [9,71]. This results in a largely empirical and reactive approach, in which treatment choices are only partially aligned with the underlying neurobiology of the neonatal brain.

Phenobarbital continues to be recommended as first-line therapy in most international guidelines, achieving electroclinical seizure control in approximately 40–50% of cases [2,9,72]. Its persistence in clinical practice is primarily driven by familiarity, availability, and long-standing experience rather than clear superiority. Indeed, phenobarbital acts through potentiation of GABAergic inhibition; however, in the neonatal brain—where GABA may exert depolarizing rather than inhibitory effects due to the predominance of NKCC1 over KCC2 transporters—its mechanism of action may be suboptimal or even paradoxical [25]. This mechanistic rationale directly motivated the clinical evaluation of bumetanide, a selective NKCC1 inhibitor, as an adjunct to phenobarbital in neonatal seizures. The European NEMO phase 1b/2 trial [73] tested bumetanide as add-on to a second loading dose of phenobarbital in term neonates with HIE-related electrographic seizures. The trial was stopped early beacause of serious adverse reactions and limited evidence for seizure reduction [73]. It did not demonstrate improved seizure control over phenobarbital alone, and—of greater concern—three of eleven surviving exposed infants developed permanent sensorineural hearing impairment, confirmed by auditory brainstem testing performed between 17 and 108 days of life [73]. This experience offers two enduring lessons that are directly relevant to the precision-neonatology framing of the present review. First, mechanistic plausibility, however biologically compelling, does not automatically translate into clinical efficacy in the developing brain: rodent models predicted neither the lack of acute clinical efficacy nor the human auditory toxicity signal. Second, the developmental NKCC1 > KCC2 imbalance remains a biologically rational therapeutic target, but its safe exploitation in neonates will require either compounds with a wider safety margin at the cochlear NKCC1, more selective brain-penetrant NKCC1 inhibitors, or interventions acting downstream of GABA-A signaling. Until such agents are validated, bumetanide cannot be recommended outside of trials in this population.

Beyond limited efficacy, phenobarbital is associated with a range of adverse effects that are particularly relevant in the neonatal population. Acute side effects include respiratory depression, hypotension, excessive sedation, and impaired feeding, often necessitating ventilatory support in critically ill neonates [29,74]. Experimental studies in animals models raise concern that phenobarbital exposure during sensitive developmental windows may induce neuronal apoptosis and interfere with synaptogenesis [75]. Observational human data have reported associations between neonatal phenobarbital exposure and adverse neurodevelopmental outcomes [76], but in this setting drug exposure is intrinsically confounded by illness severity and seizure burden, both of which are themselves strong predictors of adverse outcome. A causal independent effect of phenobarbital on human neonatal neurodevelopment has therefore not been established. Neurotoxicity should be regarded as a concern informing an individualized risk–benefit assessment, rather than as a settled clinical conclusion that should drive away from phenobarbital as first-line therapy [9,71].

Levetiracetam has emerged as a widely used alternative, largely due to its favorable safety profile and lack of apparent neurotoxicity [74,77]. Its mechanism, involving modulation of synaptic vesicle protein 2A (SV2A), is theoretically more compatible with the immature brain. In contrast to phenobarbital, levetiracetam is generally well tolerated in neonates, with minimal cardiorespiratory depression and limited sedative effects [74,77]. Reported adverse events are typically mild and include irritability, somnolence, and feeding difficulties. The pivotal NEONATAL RCT [13] directly addressed the head-to-head comparison and was stopped early after pre-planned interim analysis: approximately 80% of phenobarbital-treated neonates achieved complete electrographic seizure freedom within 24 h, versus only 28% of those treated with levetiracetam (loading 40–60 mg/kg). The magnitude of this difference is therefore well established and clearly favors phenobarbital in terms of acute efficacy. Despite this, levetiracetam continues to gain ground in real-world neonatal practice for reasons independent of acute seizure-freedom rates: a substantially more favorable cardiorespiratory safety profile, the absence of clear evidence of developmental neurotoxicity in humans (in contrast to the experimental concerns raised by phenobarbital exposure [75,76]), a more predictable intravenous pharmacokinetic profile, and ease of administration in the NICUs. Subsequent systematic reviews and meta-analyses, including the recent meta-analysis of 26 studies involving 9854 neonates [74] and the Cochrane review by Abiramalatha et al. [71], confirm phenobarbital’s superior acute efficacy while also documenting the safety advantage of levetiracetam, and emphasize the persisting absence of high-quality long-term neurodevelopmental data for either agent. Consistent with the ILAE 2023 guideline-based recommendations [9], levetiracetam should be considered a second-line option, or a context-specific first-line option when phenobarbital is contraindicated (e.g., severe cardiorespiratory instability not tolerating sedation, or where drug interactions are relevant).

**Figure 2:**
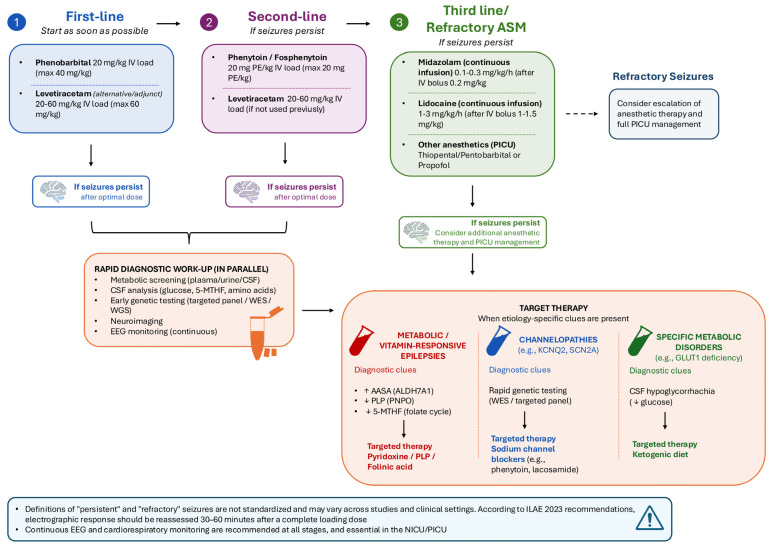
Stepwise approach of pharmacological treatment of neonatal seizures [9]. Figure Legend. ASM (antiseizure medication); IV (intravenous); PE (phenytoin equivalents); PICU (Pediatric intensive care unit); CSF (cerebrospinal fluid); 5-MTHF (5-methyltetrahydrofolate); WES (whole-exome sequencing); WGS (whole-genome sequencing); EEG (electroencephalogram); AASA (α-aminoadipic semialdehyde); PLP (pyridoxal-5′-phosphate); NICU (neonatal intensive care unit).

In clinical practice, treatment typically follows a stepwise approach, although this is not uniformly standardized and remains largely experience-based. A schematic overview of this approach is depicted in Figure 2, while dosing strategies and key pharmacological considerations are summarized in Table 5.

First-line therapy consists of phenobarbital, followed by second-line agents such as levetiracetam or phenytoin in case of persistent seizures (Figure 2, Table 5). Notably, definitions of “persistent” and “refractory” seizures are not standardized and may vary across studies and clinical settings, further contributing to variability in treatment escalation strategies (Figure 2). For the purposes of this review we adopt the following working definitions, consistent with the ILAE 2023 guideline-based recommendations [9]. Persistent seizures: any new electrographic seizure occurring after a complete loading dose of the current line of therapy, within a clinically meaningful observation window (typically 30 min). Refractory seizures: continued electrographic seizures after failure of two adequately loaded ASMs. Super-refractory seizures: seizures continuing despite a third-line agent (midazolam infusion or lidocaine). Treatment response: complete electrographic seizure freedom within 24 h of the most recent dose adjustment, in line with the NEONATAL trial endpoint [13]. Seizure burden: cumulative duration (in minutes) of electrographic seizures over a defined monitoring window, typically 24 h. These definitions are not universally adopted, and any cross-study comparison of treatment effects should explicitly state the definition used: seizure-freedom rates can change by 20–30 percentage points depending on the chosen cut-off.

Phenytoin presents several limitations in neonates, including highly variable pharmacokinetics and a narrow therapeutic window. Acute adverse effects include cardiac arrhythmias, hypotension, and local tissue injury, necessitating continuous monitoring [2,78]. In refractory cases, continuous infusions of midazolam or lidocaine may be required, particularly in intensive care settings. Midazolam carries a significant risk of respiratory depression and hypotension, often requiring mechanical ventilation, while lidocaine must be used cautiously due to potential cardiac toxicity, especially following phenytoin administration [2,79].

Importantly, in parallel with symptomatic treatment, clinicians must consider early initiation of targeted therapies in suspected metabolic or genetic epilepsies, such as pyridoxine, pyridoxal-5-phosphate, or folinic acid [9,46]. These treatments may be lifesaving but are supported mainly by limited evidence.

Among targeted metabolic therapies, folate-responsive neonatal epilepsies should be distinguished according to the underlying defect. Folinic-acid-responsive seizures, associated with ALDH7A1 variants, show substantial clinical and biochemical overlap with classic pyridoxine-dependent epilepsy and may respond to both pyridoxine and folinic acid [80]. Cerebral folate deficiency, caused by folate receptor alpha (FOLR1) variants or autoantibodies against the folate receptor, is typically treated with folinic acid supplementation [81]. In contrast, severe methylenetetrahydrofolate reductase (MTHFR) deficiency results in impaired generation of 5-methyltetrahydrofolate (5-MTHF) and disruption of methylation pathways; consequently, treatment relies primarily on methylated folate compounds together with betaine and methionine supplementation, rather than folinic acid monotherapy [82].

Ketogenic dietary therapies are not part of standard first- to third-line treatment of neonatal seizures, principally because of concerns regarding metabolic homeostasis, growth and nutrition during the neonatal period. However, the ketogenic diet has a clearly established role in specific etiologies that can manifest in the neonatal window: it is the treatment of choice in GLUT1 deficiency syndrome (SLC2A1), and it is increasingly used in pyruvate dehydrogenase complex deficiency [83,84]. In addition, case series have reported its use as last-line therapy in selected neonates with super-refractory status epilepticus, in highly specialized centers and under close metabolic supervision [85]. The decision to start a ketogenic diet in a neonate should be confined to centers with combined pediatric neurology and metabolic expertise and should follow a clear etiological hypothesis rather than be used as a generic anticonvulsant strategy.

Cannabidiol is established as adjunctive therapy in selected older-pediatric drug-resistant epilepsy syndromes (Dravet, Lennox–Gastaut, tuberous-sclerosis-associated epilepsy) [86,87], but no neonatal-specific efficacy or safety data are currently available. Cannabidiol should therefore be considered experimental in neonates and is not part of standard neonatal seizure management. Any neonatal use should be confined to research protocols with appropriate safety monitoring. Its potential role in severe neonatal epilepsies remains exploratory and further underscores the expanding use of off-label therapies in the absence of neonatal-specific evidence.

A major unresolved issue is the lack of clear criteria for treatment escalation and switching. Decisions are frequently based on electrographic seizure burden, clinical severity, and response latency rather than standardized thresholds. The widespread use of cEEG monitoring has further revealed a high prevalence of subclinical seizures, complicating both diagnosis and treatment [2,9].

Overall, the pharmacological management of neonatal seizures exemplifies an “evidence gap” scenario, in which widespread clinical practices are only partially supported by high-quality data. This gap is further compounded by developmental pharmacology, etiological heterogeneity, and the delicate balance between efficacy and potential neurotoxicity. As a result, current treatment approaches remain largely empiric and reactive rather than mechanism-driven and predictive. Bridging this gap will require neonatal-specific RCTs, improved biomarkers, and integration of precision medicine approaches.

## 6. Long-Term Outcome

### 6.1. Long-Term Outcomes After Acute Symptomatic Neonatal Seizures

Long-term neurodevelopmental outcome in neonatal seizures has traditionally been considered primarily dependent on the underlying etiology, with conditions such as HIE, intracranial hemorrhage, and genetic disorders carrying markedly different prognostic implications [7,88,89].

Among these, acute symptomatic causes—particularly hypoxic–ischemic injury—remain the strongest predictors of adverse cognitive and motor outcomes, including cerebral palsy and epilepsy [29,90].

However, this etiology-centered view has progressively been challenged by evidence suggesting that early neurophysiological markers, especially EEG features, provide additional and independent prognostic information [91,92].

Abnormal background activity, increased discontinuity, and the absence of normal SWC on EEG and aEEG have been consistently associated with adverse neurodevelopmental outcomes, independently of the underlying etiology [53,93,94].

In contrast, the presence of a more continuous and reactive EEG background pattern is generally associated with more favorable neurodevelopmental outcomes [94,95].

Seizure burden has also emerged as an important prognostic factor, with higher cumulative seizure duration being associated with worse cognitive and motor outcomes in observational cohorts [88,96], with a dose–response relationship that persists after adjustment for measured etiology and severity. However, residual confounding by unmeasured severity of the underlying encephalopathy cannot be excluded in any observational design: etiology and seizure burden are strongly correlated, so adjustment for measured covariates leaves residual confounding by unmeasured severity. A causal contribution of seizures to secondary brain injury independent of the underlying insult has therefore not been established by randomized or quasi-experimental evidence in human neonates. Experimental models [26] provide mechanistic plausibility for such an independent contribution but cannot be directly transposed to the human neonate. We present seizure burden as a prognostic marker of disease severity with biologically plausible (but not proven) modifier effects, rather than as a confirmed causal mediator.

In this context, early recognition and treatment of neonatal seizures have been proposed as potentially modifiable factors that may influence outcome, although high-quality evidence from RCTs remains limited [95].

Importantly, electroclinical uncoupling following antiseizure medication administration may lead to an underestimation of ongoing seizure activity, potentially delaying adequate treatment and influencing outcome [53,97].

More recently, increasing attention has been directed toward the identification of early biomarkers that may refine prognostic stratification beyond clinical and conventional EEG parameters. These include advanced neuroimaging techniques and quantitative EEG approaches, which may provide additional insight into both the extent of brain injury and underlying pathophysiological mechanisms [90,94].

Taken together, these findings support a shift from a purely etiology-driven model toward integrated predictive approaches that combine clinical variables, EEG features, seizure burden, and treatment-related factors [7,88,96].

Such multidimensional models may improve early prognostic accuracy and help guide more individualized therapeutic strategies in neonatal seizures [7].

Two concepts borrowed from adult epileptology—the pre-ictal state and postictal generalized EEG suppression (PGES)—have received limited attention in neonatal epileptology. Research on pre-ictal prediction in neonates remains in its infancy: most published work has focused on automated seizure detection (e.g., the ANSeR machine-learning algorithm [98]) and on the prognostic value of early EEG background features, such as automated background classification in neonates with HIE undergoing therapeutic hypothermia [56,99], rather than on the identification of a reproducible pre-ictal signature. Similarly, reliable neonatal-specific data on PGES are currently lacking, and EEG patterns following neonatal seizures often overlap with the persistent background abnormalities of the underlying encephalopathy, making discrimination between postictal and baseline activity particularly challenging [100]. These limitations define an important research agenda, including the development and validation of neonatal seizure-prediction algorithms with clinically meaningful lead times, acceptable false-alarm rates, and integration into NICUs workflows, as well as prospective characterization of postictal EEG dynamics in neonatal populations. Until such data become available, the hypothesis that pre-ictal seizure prediction or modification of postictal EEG suppression could improve long-term neurodevelopmental outcomes in neonates remains unproven [2].

### 6.2. Long-Term Outcomes in Neonatal-Onset Genetic Epilepsies and DEEs

In neonatal-onset genetic epilepsies and DEEs, the developmental component of the disease frequently dominates the long-term phenotype, irrespective of seizure control [39]. In KCNQ2-DEE, STXBP1-DEE, and CDKL5 deficiency disorder, neurodevelopmental impairment is often substantial even when seizures are adequately controlled, whereas in self-limited neonatal KCNQ2-related epilepsy development may remain normal [11,16,39]. Seizure freedom is therefore an important but insufficient outcome measure in this group, and prognostic counselling should integrate the molecular diagnosis, the functional consequence of the variant, and the availability of targeted therapies [2,39].

This distinction highlights a fundamental difference between (i) acute symptomatic neonatal seizures, in which outcome is determined primarily by the severity of the underlying brain injury, with seizure burden acting as an additional prognostic modifier, and (ii) neonatal-onset genetic epilepsies and DEEs, in which the developmental consequences of the underlying disorder often represent the dominant determinant of long-term outcome [2,11]. This conceptual separation has important implications for the design and interpretation of future neonatal seizure trials, as pooled analyses that fail to distinguish these populations may obscure clinically meaningful treatment effects within either group.

## 7. Limitations and Biases in Neonatal Epilepsy: Bridging Evidence and Practice

Neonatal epilepsy represents a paradigmatic example of a field in which clinical decision-making is frequently shaped by uncertainty, heterogeneity, and implicit assumptions rather than robust evidence. Despite major advances in neurophysiology, genetics, and intensive care monitoring, several critical biases and limitations continue to influence both clinical practice and research, often in ways that are insufficiently acknowledged [29,71].

One of the most debated and unresolved issues concerns the threshold for treatment and the degree of therapeutic aggressiveness. While electrographic seizures are increasingly recognized as clinically relevant—even in the absence of overt clinical manifestations—the extent to which they independently contribute to brain injury remains uncertain [7,101]. In many cases, particularly in acute symptomatic seizures, it is difficult to disentangle the impact of the underlying brain insult from that of the seizures themselves. As a result, current practice often favors early and aggressive treatment, driven by the assumption that reducing seizure burden will improve neurodevelopmental outcomes. However, this assumption is not uniformly supported by high-quality evidence and must be balanced against the potential neurotoxicity of ASMs in the developing brain [71,75]. This creates a fundamental therapeutic tension between overtreatment and undertreatment.

A second major limitation emerges in the context of severe genetic epilepsies. The increasing availability of rapid genomic diagnostics has transformed early etiological identification in neonatal epilepsy [102,103]. However, in many of these conditions, seizures represent only one component of a broader developmental encephalopathy driven by intrinsic biological mechanisms. In such cases, conventional ASMs often have limited efficacy, and seizure control does not necessarily translate into improved neurodevelopmental outcomes [103]. This highlights a critical limitation of current therapeutic approaches: the persistence of a seizure-centric model in conditions where seizures may be an epiphenomenon rather than the primary driver of disease.

From a research perspective, much of the available evidence in neonatal epilepsy is derived from studies conducted in tertiary referral centers. While these settings provide access to advanced monitoring and specialized expertise, they also introduce significant selection bias. Neonates admitted to tertiary centers are more likely to present with severe, complex, or refractory conditions, leading to an overrepresentation of high-risk phenotypes in the literature [29,104]. Consequently, the epidemiology, treatment responses, and outcomes reported in these cohorts may not be generalizable to the broader neonatal population.

Conversely, milder or self-limited forms of neonatal seizures are likely under-represented in current research. These cases may not require intensive care admission or cEEG monitoring and are therefore less likely to be captured in prospective studies. This imbalance contributes to a distorted perception of neonatal epilepsy as predominantly severe and pharmacoresistant, potentially influencing both clinical expectations and treatment strategies [104].

A further methodological challenge concerns the limited semiological resolution between etiologies at the neonatal bedside. Most causes of neonatal seizures—including HIE, perinatal arterial ischemic stroke, intracerebral hemorrhage, CNS infections, malformations of cortical development, IEMs, and monogenic channelopathies—converge on a comparatively narrow electroclinical repertoire characterized by subtle and multifocal clinical signs, frequent electrographic-only events, and background abnormalities ranging from discontinuity to BS. As a result, electroclinical features are rarely sufficient on their own to identify a specific etiology, with the exception of a few high-yield patterns such as the sequential seizures described in KCNQ2-related epilepsy [16]. The diagnostic value of EEG therefore lies primarily in seizure detection, seizure-burden quantification, and assessment of background brain function, whereas etiological resolution depends on the integration of perinatal history, neuroimaging, metabolic investigations, and increasingly rapid genomic testing.

Finally, the development of high-quality RCTs in neonates remains inherently challenging. Ethical constraints, small and heterogeneous patient populations, rapidly evolving clinical conditions, and the need for urgent intervention all limit the feasibility of traditional trial designs [71,105]. In addition, the lack of standardized definitions—particularly for concepts such as seizure burden, treatment response, and refractoriness—further complicates study design and interpretation. As a result, much of neonatal epilepsy care continues to rely on low- to moderate-quality evidence, reinforcing the gap between clinical practice and scientific validation.

Taken together, these considerations suggest that the limitations of neonatal epilepsy care are not solely technological or pharmacological, but conceptual. The field continues to rely on frameworks that may be insufficiently adapted to the unique biology of the developing brain. Recognizing these biases is a necessary step toward a more nuanced and biologically grounded approach, in which treatment decisions are better aligned with etiology, developmental context, and long-term outcomes.

## 8. Future Perspectives: Towards Precision Neonatology in Epilepsy

Over the next decade, the management of neonatal seizures is expected to evolve from a predominantly reactive and descriptive approach toward a model of care that is continuous, predictive, and biologically stratified. This transition is being driven by the convergence of three major technological domains: AI applied to neonatal EEG, rapid genomic diagnostics integrated into neonatal intensive care workflows, and multimodal neuromonitoring combining electrophysiological, hemodynamic, metabolic, and imaging-derived data. Increasingly, these approaches are being conceptualized not as isolated tools, but as interconnected components of a precision neurocritical care framework tailored to the developing brain [14,106].

Among the themes recurring across recent literature, AI emerged as a particularly active area of development, with applications ranging from automated seizure detection and EEG interpretation to seizure-burden quantification and clinical decision-support systems [98,99]. Machine-learning and deep-learning models have demonstrated robust performance in automated seizure detection, including the identification of electrographic-only seizures that are frequently missed in routine clinical practice [98]. However, the field is rapidly moving beyond simple event detection toward quantitative EEG analysis, including automated background classification, seizure burden estimation, and extraction of early prognostic features from continuous recordings [99]. This evolution reflects a broader conceptual shift: EEG is increasingly being regarded as a dynamic biomarker of brain function and injury. Early EEG features—such as background suppression, impaired SWC, and high seizure burden—have been consistently associated with adverse neurodevelopmental outcomes, particularly in neonatal encephalopathy [99,107].

In parallel, advances in neuroimaging are expanding the landscape of early biomarkers of brain injury. Diffusion-weighted imaging remains central to the early identification of hypoxic–ischemic injury, while emerging connectome-based approaches, including diffusion tensor imaging and functional MRI, provide insights into network organization and dysmaturation [23,108]. Importantly, the integration of electrophysiological and imaging biomarkers has been shown to improve the prediction of neurodevelopmental outcomes compared with single-modality approaches, supporting a multimodal strategy for early prognostication [108].

A further and increasingly relevant dimension is represented by circulating serum biomarkers reflecting neuronal injury, glial activation, and oxidative stress. Biomarkers such as neuron-specific enolase, S100B, glial fibrillary acidic protein, and ubiquitin carboxy-terminal hydrolase L1 have been associated with the severity of neonatal brain injury and subsequent neurodevelopmental outcomes [57]. In addition, biomarkers of oxidative stress and antioxidant imbalance have been shown to reflect ongoing cellular injury and secondary damage cascades in both preterm and full-term neonates, underscoring the role of oxidative mechanisms in neonatal brain injury [57]. As highlighted in recent comprehensive reviews, circulating biomarkers offer the advantage of minimally invasive and repeatable assessment, potentially enabling longitudinal monitoring of brain injury evolution and complementing electrophysiological and imaging data in early diagnosis and prognostic stratification [57]. Table 6 summarizes the principal biomarkers studied to date, their biological significance, and their proposed clinical applications. To date, no circulating biomarker has demonstrated sufficient specificity for seizure activity per se in the neonatal period. The principal value of these markers lies in assessing the severity and evolution of the underlying brain injury. Accordingly, they should be regarded as complementary to EEG monitoring and neuroimaging rather than as diagnostic substitutes for neonatal seizure detection and characterization [57,108,109].

A complementary translational line concerns the oxidative-stress/antioxidant-defense axis. Preclinical studies in epilepsy models suggest that NOX2/NADPH oxidase–mediated neuroinflammation contributes to seizure-related neuronal injury, and that activation of the Keap1–Nrf2 antioxidant response pathway and modulation of thioredoxin-related systems can attenuate this injury in vitro and in adult-rodent in vivo models [110]. In the neonatal context, oxidative-stress biomarkers (8-isoprostane, advanced oxidation protein products, total antioxidant capacity) are altered in HIE and prematurity-related brain injury [57]. However, NOX2-, Nrf2- and thioredoxin-targeted interventions have not been tested in human neonates with seizures, and their clinical translation will require neonatal-specific safety and pharmacokinetic data [57]. These pathways therefore represent promising but currently preclinical research directions, underscoring the need for caution when extrapolating therapeutic findings from adult experimental models to the developing human brain [73].

Within this framework, multimodal neuromonitoring is evolving from parallel data acquisition toward true integration of physiological signals. The combination of cEEG with near-infrared spectroscopy (NIRS), cardiorespiratory monitoring, and advanced neuroimaging techniques enables simultaneous assessment of electrical activity, cerebral perfusion, oxygenation, and metabolic state [106,109]. Importantly, emerging approaches aim to integrate these heterogeneous data streams into composite biomarkers capable of capturing dynamic injury trajectories and identifying high-risk infants before clinical deterioration becomes evident. In this context, AI is likely to play a central role, enabling the identification of complex, multidimensional patterns that are not accessible through conventional analysis.

Rapid genomic diagnostics represent a second major axis of transformation. Rapid whole-exome and whole-genome sequencing can now provide molecular diagnoses within days—and in some settings within 24–48 h—allowing early identification of genetic etiologies in critically ill neonates [111]. This is particularly relevant in neonatal epilepsies, where early molecular diagnosis may directly inform treatment decisions, including the use of targeted therapies and precision pharmacological approaches [111,112]. Beyond diagnostic yield, the clinical impact of rapid sequencing increasingly includes changes in management, improved prognostic counseling, and reduction in diagnostic uncertainty during a critical window of brain development [111].

**Figure 3:**
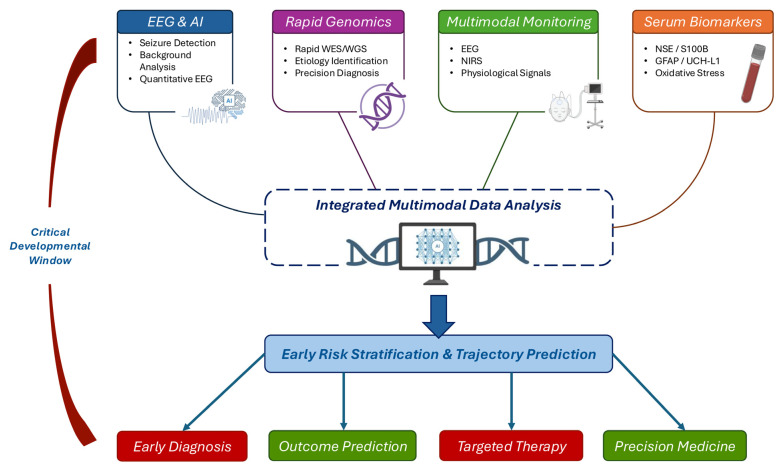
Future model of neonatal seizure care: integration of multimodal biomarkers for prediction and precision treatment. Figure Legend. EEG (electroencephalogram); AI (artificial intelligence); WES (whole-exome sequencing); WGS (whole-genome sequencing); NIRS (near-infrared spectroscopy); NSE (Neuron-Specific Enolase); S100B (S100 Calcium-Binding Protein B); GFAP (Glial Fibrillary Acidic Protein); UCH-L1 (Ubiquitin C-terminal Hydrolase L1).

Taken together, these advances suggest that neonatal seizure care is moving toward a model in which detection, diagnosis, and prediction are integrated within a unified framework, as summarized in Figure 3.

Rather than focusing solely on the presence of seizures, future clinical approaches will likely incorporate multidimensional assessment of brain function, structure, and biology. In such a model, EEG will provide continuous functional monitoring, imaging will define structural and network-level injury, circulating biomarkers will reflect underlying cellular and molecular processes, and genomics will identify etiological substrates. The integration of these domains—supported by AI—has the potential to enable early identification of infants at highest risk of adverse neurodevelopmental outcomes and to guide individualized, mechanism-based therapeutic strategies.

Despite these promising developments, significant challenges remain, including the need for large-scale validation, standardization of data acquisition and interpretation, integration into clinical workflows, and equitable access to advanced technologies. More specifically: AI-EEG tools require external validation across populations and recording systems, with explicit characterization of false-alarm rates in continuous monitoring and of generalizability across centers—only the ANSeR multicenter RCT [88] has provided RCT-level evidence in neonates to date. Rapid neonatal whole-exome sequencing (WES)/whole-genome sequencing (WGS) has demonstrated diagnostic yield but the prospective evidence on its impact on management decisions in critically ill neonates is still limited [111,112]. Circulating biomarkers reflect the severity and evolution of underlying brain injury but are not seizure-specific; their integration into clinical decision-making requires assays with neonatal reference ranges and prospective outcome studies [57]. NIRS and multimodal integration are in earlier phases of validation. Cost-effectiveness assessment and workflow integration are largely missing from the current evidence base. Therefore, the most realistic vision for the next decade is not full automation, but a transition toward earlier, more standardized, and more biologically informed care. Ultimately, the convergence of AI, genomics, multimodal monitoring, and biomarker-based prediction may transform neonatal epilepsy from a field centered on seizure detection into one focused on the anticipation of brain injury and the preservation of neurodevelopment.

A final and often-overlooked limitation concerns the geographical concentration of the evidence base. Most cohort studies, RCTs, and clinical guidelines originate from high-income-country settings with continuous neurophysiology coverage, multichannel cEEG capability, rapid genomic diagnostics, and routine implementation of therapeutic hypothermia. In low- and middle-income countries (LMICs), the operational reality is frequently different. aEEG, which requires less specialized neurophysiology expertise and can be operated at the bedside by neonatologists, often plays a more prominent diagnostic role than cEEG, and the combination of bedside aEEG, on-demand cEEG, and remote expert review may represent the most realistic neuromonitoring model in many LMICs NICUs. Rapid WES/WGS remains unavailable or accessible only in selected centers in many LMICs settings, and etiological diagnosis therefore continues to rely primarily on clinical context, EEG findings, and neuroimaging when available. Therapeutic hypothermia for HIE is also not yet universally implemented. The recommendations distilled in this review should therefore be interpreted with the explicit recognition that they largely reflect evidence generated in high-income settings. Adaptation to local resources and the development of feasible diagnostic and therapeutic pathways for LMICs environments remain important priorities for future research and implementation efforts.

## 9. Conclusions

Neonatal epilepsy represents a complex and evolving field at the intersection of developmental neurobiology, heterogeneous etiologies, and rapidly advancing diagnostic technologies. While significant progress has been made in seizure detection and etiological characterization—particularly through cEEG monitoring and genomic diagnostics—the translation of these advances into effective and individualized treatments remains limited.

A key challenge lies in the persistent gap between knowledge and practice. Current therapeutic strategies are still largely empirical, with limited neonatal-specific evidence and continued reliance on historical treatments such as phenobarbital. At the same time, emerging data challenge the assumption that seizure suppression alone is sufficient to improve long-term outcomes, particularly in the context of severe genetic and developmental epilepsies.

This review highlights a fundamental conceptual shift: neonatal epilepsy should not be viewed solely as a disorder of seizures, but as a disorder of the developing brain. From this perspective, seizures represent only one dimension of a broader and dynamic pathological process. Looking forward, the integration of electrophysiological, genetic, imaging, and molecular data—supported by advances in AI—offers the opportunity to move toward a more precise and biologically informed model of care. However, this transition remains incomplete and will require not only technological innovation but also the development of neonatal-specific evidence, standardized frameworks, and etiology-driven therapeutic strategies.

Main findings of this narrative review can be summarized as follows: (i) The neonatal brain is a developmentally constrained system; the immaturity of its connectivity, the depolarizing action of GABA in the early postnatal period and the still-evolving SWC shape an electroclinical distinct seizure phenotype that cannot be approached with frameworks borrowed from older patients; (ii) Most neonatal seizures are eletrografic-only, and clinical observation systematically underestimates true seizure burden; cEEG remains the gold standard for detection and quantification, while aEEG retains a complementary role for continuous bedside screening, particularly in centers without continuous neurophysiology coverage; (iii) Etiology—chiefly HIE, perinatal stroke, intracranial hemorrhage, malformations of cortical development, IEMs and monogenic neonatal-onset DEEs—is the strongest determinant of long-term outcome, while seizure burden acts as an independent and potentially modifiable prognostic modifier; (iv) Phenobarbital retains an evidence-based advantage in acute electrographic seizure control; levetiracetam offers a more favorable safety profile but inferior acute efficacy; (v) Rapid genomic diagnostics, AI-assisted EEG analysis and multimodal neuromonitoring are converging toward a precision-neonatology framework; their translation into routine practice requires external validation, standardization, cost-effectiveness assessment and equitable access.

Ultimately, the goal of neonatal epilepsy care should extend beyond seizure control to the preservation and optimization of neurodevelopmental outcomes.

## Figures and Tables

**Table 1:** Major neonatal-onset genetic epilepsies: genotype–phenotype correlations and therapeutic implications.

Gene	Inheritance	Typical Age of Onset	Seizure Pattern	EEG Features	Developmental Outcome	Treatment Considerations
**KCNQ2** [11,16,35,41]	AD (de novo most common)	First days of life	Sequential seizures (tonic posturing, clonic movements, autonomic signs)	Electrical decrement followed by rhythmic spikes; burst-suppression in DEE forms	Variable, from self-limited neonatal seizures with normal development to severe DEE	Sodium-channel blockers frequently effective, particularly in neonatal-onset phenotypes and selected GoF variants; LoF/dominant-negative variants often show poorer response; rapid genetic diagnosis may influence treatment decisions
**KCNQ3** [11,41]	AD	Neonatal period	Sequential seizures reported; phenotype overlaps with KCNQ2	Similar to KCNQ2	Usually milder than KCNQ2-associated disease	Therapeutic recommendations largely extrapolated from KCNQ2; evidence remains limited
**SCN2A** [36,37,38,39,40]	AD (de novo most common)	First hours–days (GoF) vs. later infancy (LoF)	Focal or sequential seizures in GoF forms; heterogeneous phenotypes in LoF variants	Multifocal seizures; abnormal background activity	GoF variants often associated with neonatal epilepsy; LoF variants frequently associated with autism spectrum disorder and intellectual disability	Sodium-channel blockers often effective in GoF variants; may be ineffective or detrimental in LoF variants; precision therapies including antisense oligonucleotides are under investigation
**STXBP1** [11,42]	AD (de novo most common)	Neonatal to early infantile period	Tonic seizures, multifocal seizures	Burst-suppression common; multifocal epileptiform discharges	Severe developmental impairment frequently exceeds seizure burden	Response to conventional ASMs is variable; targeted therapies under development
**CDKL5** [2,43]	X-linked dominant (female predominance)	First weeks–months of life	Multifocal seizures with evolution to epileptic spasms	Multifocal discharges; hypsarrhythmia in later stages	Severe DEE	Conventional ASMs often provide limited benefit; ganaxolone represents the first disease-specific approved therapy
**TSC1/TSC2** [11,44]	AD; mosaicism possible	Usually first months of life (rarely neonatal)	Focal seizures, often evolving to infantile spasms	Multifocal discharges; hypsarrhythmia may develop	Variable; intellectual disability and autism spectrum disorder common in severe forms	Vigabatrin first-line therapy; mTOR inhibitors (everolimus) represent targeted treatment
**PAFAH1B1 (LIS1)** [11,45]	AD (de novo most common)	Neonatal to early infantile period	Multifocal, frequently drug-resistant seizures	Multifocal discharges; severely abnormal background activity	Severe global developmental impairment	Symptomatic treatment; response to ASMs generally limited
**ALDH7A1** (Pyridoxine-dependent epilepsy) [9,46]	AR	First hours–days of life	Refractory multifocal seizures, apnea, encephalopathy	Burst-suppression frequently reported	Outcome improves with early recognition, although cognitive impairment may persist	Empirical pyridoxine trial recommended in neonatal seizures of unknown etiology; long-term treatment includes pyridoxine and lysine-reduction therapies
**PNPO** [2,3,4,5,6,7,8,9]	AR	First days of life	Refractory multifocal seizures	Burst-suppression common	Variable; cognitive impairment frequent	Pyridoxal-5′-phosphate is usually preferred; pyridoxine may show partial efficacy in selected patients

Table legend: EEG (electroencephalography); AD (autosomal dominant); DEE (developmental and epileptic encephalopathy); GoF (gain-of-function); LoF (loss-of-function); ASM (antiseizure medication); mTOR (mechanistic target of rapamycin); AR (autosomal recessive).

**Table 2:** Electroclinical features and therapeutic implications of major etiological categories of neonatal seizures [9,11,16,29,35,36,46,56].

Etiological Category	Typical Age at Onset	Seizure Semiology	EEG Features	Key Diagnostic Clues	First-Line Therapeutic Implication
HIE	First 24–72 h	Subtle, clonic, often multifocal; electrographic-only seizures common	Abnormal background, discontinuity, low voltage or burst-suppression; variable seizure burden	Perinatal sentinel event, low Apgar score, umbilical cord acidosis, MRI pattern of hypoxic–ischemic injury	Treat acute symptomatic seizures; phenobarbital remains the usual first-line ASM; optimize neurocritical care and therapeutic hypothermia when indicated
Perinatal arterial ischemic stroke	First 24–72 h	Focal clonic seizures, often unilateral	Focal electrographic seizures with localised slowing	Focal arterial-territory lesion on MRI; vascular or cardiac risk factors	Treat focal acute symptomatic seizures, usually with phenobarbital first-line ASM; consider etiology-directed stroke evaluation
Intracranial hemorrhage	First days	Focal or multifocal seizures; may be subtle	Focal slowing, asymmetric background, focal or multifocal seizures	Cranial ultrasound and MRI evidence of haemorrhage	Treat acute symptomatic seizures; manage haemorrhage-related complications and underlying coagulopathy when present
CNS infection	First days–weeks	Multifocal clonic seizures or status epilepticus; encephalopathy common	Diffuse slowing, multifocal epileptiform abnormalities, variable seizure burden	CSF abnormalities, blood inflammatory markers, microbiological testing.	Treat seizures and start urgent antimicrobial/antiviral therapy according to suspected pathogen
Channelopathies (KCNQ2, KCNQ3, SCN2A, STXBP1, CDKL5)	First hours–days or early infancy	Sequential seizures (KCNQ2/SCN2A), tonic (STXBP1), multifocal/spasms (CDKL5)	Burst suppression or multifocal epileptiform activity depending on genotype	Early onset without clear acute insult; family history; rapid genetic testing	Early genetic diagnosis; consider genotype-guided treatment, including sodium channel blockers in KCNQ2/SCN2A gain-of-function, ganaxolone in CDKL5, supportive in STXBP1
Malformations of cortical development	First months	Focal or multifocal seizures; evolution to infantile spasms may occur	Multifocal epileptiform discharges; focal abnormalities depending on lesion	MRI evidence of cortical malformation, tubers, lissencephaly, or other structural anomaly	ASM choice guided by structural substrate and seizure type; vigabatrin first-line for infantile spasms in TSC
Inborn errors of metabolism	First hours–days	Refractory multifocal seizures often with apnoea, epileptic spasms, or status epilepticus	Multifocal epileptiform abnormalities; background may be severely abnormal	Metabolic acidosis, abnormal amino acids/organic acids, hypoglycaemia, hyperammonaemia, or vitamin/cofactor responsiveness	Urgent metabolic testing. Empirical pyridoxine trial; pyridoxine in PDE, PLP in PNPO, dietary/cofactor management in others

Table Legend. EEG (electroencephalography); HIE (hypoxic–ischemic encephalopathy); ASM (antiseizure medication); MRI (magnetic resonance imaging); CNS (central nervous system); CSF (cerebrospinal fluid); TSC (tuberous sclerosis complex); PDE (pyridoxine-dependent epilepsy); PLP (pyridoxal-5′-phosphate); PNPO (pyridox(am)ine-5′-phosphate oxidase deficiency).

**Table 3:** Normal electroencephalographic features in neonates according to gestational age [22,55,62,63].

Gestational Age	Background Activity	Continuity	Synchrony	Characteristic Features	Sleep–Wake Organization
<28 weeks	Discontinuous, low organization	Markedly discontinuous (long interburst intervals)	Poor	Bursts of mixed frequencies	Absent or not distinguishable
28–32 weeks	Discontinuous but more organized	Reduced interburst intervals	Emerging synchrony	More structured bursts	Immature, beginning differentiation
32–34 weeks	Increasing organization	Moderately discontinuous → approaching continuity	Improved synchrony	Delta brushes prominent	Early cycling appears
34–37 weeks	More organized background	Largely continuous (especially in active sleep)	Good synchrony	Delta brushes decrease, more defined patterns	Clearer sleep-state differentiation
≥37 weeks	Well-organized background	Predominantly continuous	Symmetric and synchronous	Encoches frontales, temporal theta activity	Well-defined sleep–wake cycling

**Table 4:** Comparison between aEEG and conventional EEG in neonatal seizure monitoring.

	aEEG	Conventional EEG
Number of channels	Limited (1–2 channels)	Multichannel (full montage)
Ease of use	High (bedside, real-time)	Requires neurophysiology expertise
Availability	Widely available in NICUs	Limited, resource-dependent
Continuous monitoring	Yes	Yes
Background assessment	Good (trend-based)	Detailed and comprehensive
Seizure detection sensitivity	Moderate (~40–60%)	High (gold standard)
Detection of focal/brief seizures	Limited	Reliable
Quantification of seizure burden	Approximate	Accurate
Susceptibility to artifacts	Moderate	Lower (with expert interpretation)
Clinical role	Screening and trend monitoring	Diagnostic confirmation

Table Legend. aEEG (Amplitude-integrated electroencephalography); EEG (Electroencephalogram); NICUs (neonatal intensive care units).

**Table 5:** Pharmacological treatment of neonatal seizures.

Drug	Line	Loading Dose	Maintenance Dose	Key Considerations	Adverse Effects
**Phenobarbital**	1st line	20 mg/kg (up to 40 mg/kg)	3–4 mg/kg/day	Limited efficacy	Respiratory depression, hypotension, sedation, neuronal apoptosis, cognitive impairment
**Levetiracetam**	1–2nd line	40–60 mg/kg	20–60 mg/kg/day	Good safety profile	Irritability, somnolence, limited long-term data
**Phenytoin/Fosphenytoin**	2nd line	20 mg/kg	4–8 mg/kg/day	Variable PK	Arrhythmias, hypotension, tissue injury
**Midazolam**	3rd line	0.1–0.2 mg/kg	infusion	ICU use	Respiratory depression, hypotension, tolerance
**Lidocaine**	Refractory	2 mg/kg	infusion	Narrow therapeutic window	Cardiac arrhythmias
**Pyridoxine**	Targeted	100 mg IV	—	Life-saving in PDE	Apnea, hypotension
**Pyridoxal-5-phosphate**	Targeted	variable	—	PNPO deficiency	Limited data
**Folinic acid**	Targeted	variable	—	Metabolic epilepsy	Limited data

Table Legend. PK (Pharmacokinetics); ICU (Intensive Care Unit); PDE (Pyridoxine-Dependent Epilepsy); PNPO (pyridox(am)ine-5′-phosphate oxidase).

**Table 6:** Circulating biomarkers investigated in neonatal brain injury and neonatal seizures [57,108,109].

Biomarker	Cellular Source/Molecular Role	Reported Clinical Correlate	Specificity for Seizure Activity	Current Clinical Utility
Neuron-Specific Enolase	Neuronal cytoplasmic glycolytic enzyme	Elevated in HIE, perinatal arterial ischemic stroke, severe neonatal encephalopathy; associated with extent of neuronal injury	Low; reflects neuronal injury rather than seizure activity per se	Research and prognostic biomarker
S100B	Astroglial calcium-binding protein	Elevated in HIE, IVH, and other neonatal brain injuries	Low; influenced by astroglial activation and extracerebral sources	Research and prognostic biomarker
Glial Fibrillary Acidic Protein	Astrocytic intermediate filament protein	Elevated in HIE and severe neonatal encephalopathy; associated with astroglial injury	Low; not specific for seizures	Experimental biomarker
Ubiquitin C-terminal Hydrolase L1	Neuronal deubiquitinating enzyme	Elevated in acute neuronal injury, particularly HIE	Low; reflects neuronal damage rather than ictal activity	Experimental biomarker
Neurofilament Light Chain	Axonal cytoskeletal protein	Correlates with severity of neonatal brain injury and adverse neurodevelopmental outcome	Low; not seizure-specific	Emerging prognostic biomarker
Oxidative stress markers (8-isoprostane, advanced oxidation protein products, total antioxidant capacity)	Markers of redox imbalance and oxidative injury	Correlate with severity of HIE and prematurity-related brain injury	Low; reflect systemic and cerebral oxidative stress rather than seizures	Research only

Table legend. HIE (hypoxic–ischemic encephalopathy); IVH (intraventricular hemorrhage).

## Data Availability

No new data were created or analyzed in this study. Data sharing is not applicable to this article.

## Supplementary Materials

The following supporting information can be downloaded at https://www.mdpi.com/article/10.3390/brainsci16060628/s1. Table S1: Scale for the Assessment of Narrative Review Articles [17].

## Abbreviations

The following abbreviations are used in this manuscript:

AI	Artificial intelligence
ALDH7A1	Aldehyde dehydrogenase 7 family member A1
ASMs	Antiseizure medications
aEEG	Amplitude-integrated electroencephalogram
BS	Burst suppression
cEEG	Continuous electroencephalogram
CLV	Continuous low voltage
CNS	Central nervous system
CNV	Continuous normal voltage
DEEs	Developmental and epileptic encephalopathies
DNV	Discontinuous normal voltage
EEG	Electroencephalogram
FOLR1	Folate receptor alpha
FT	Flat trace
GABA	γ-aminobutyric acid
GABAergic	γ-aminobutyric acid-mediated
HIE	Hypoxic–ischemic encephalopathy
IEMs	Inborn errors of metabolism
ILAE	International League Against Epilepsy
LMICs	Low- and middle-income countries
MRI	Magnetic resonance imaging
MTHFR	Methylenetetrahydrofolate reductase
NICUs	Neonatal intensive care units
NIRS	Near-infrared spectroscopy
PGES	Postictal generalized electroencephalogram suppression
RCTs	Randomized controlled trials
SANRA	Scale for the Assessment of Narrative Review Articles
SV2A	Synaptic vesicle protein 2A
SWC	Sleep–wake cycling
5-MTHF	5-methyltetrahydrofolate
