# Supplementary material for: Neonatal Epilepsy: Beyond Seizures in a Developing Brain—A Narrative Review
Source: Brain Sci. 2026 Jun 11;16(6):628. doi: 10.3390/brainsci16060628 (PMC13297448; doi:10.3390/brainsci16060628)
Supplement: Supplementary file 1 [file brainsci-16-00628-s001.zip › brainsci-4324356-supplementary.pdf]

Table S1. Scale for the Assessment of Narrative Review Articles [17].

SANRA Domain	Justification for Scoring	Score
1. Justification of the importance of the review for the readership	The review addresses a clinically relevant topic with ongoing developments in diagnosis, management, and research. The rationale and significance of the topic are clearly outlined in the Introduction.	2
2. Statement of concrete aims or formulation of questions	The objectives and scope of the review are explicitly stated and consistently addressed throughout the manuscript.	2
3. Description of the literature search	The Methods section describes the databases consulted, search terms, article selection process, and inclusion approach used to identify relevant literature.	2
4. Referencing	Statements and conclusions are supported by appropriate references, including primary studies, reviews, and relevant guidelines where applicable.	2
5. Scientific reasoning	The review critically evaluates the available literature, discusses areas of uncertainty, and presents current evidence within an appropriate scientific context.	2
6. Appropriate presentation of data	The information is presented in a structured and logical manner, supported by tables and figures where appropriate to facilitate understanding.	2
**Total Score**		**12**
